# Binary meta-hologram for a reconfigurable holographic metamaterial antenna

**DOI:** 10.1038/s41598-020-65458-3

**Published:** 2020-05-22

**Authors:** Ruey-Bing (Raybeam) Hwang

**Affiliations:** 1 Institute of Communications Engineering, College of Electrical and Computer Engineering, National Chiao Tung University, Hsinchu, 30050 Taiwan; 2 Center for mmWave Smart Radar Systems and Technologies, National Chiao Tung University, Hsinchu, 30050 Taiwan

**Keywords:** Electrical and electronic engineering, Computational methods

## Abstract

This research reports a design method for synthesizing the binary meta-hologram pattern implemented in a leaky waveguide that can radiate the feeding signal toward a prescribed direction. In fact, the obtained pattern is not always a uniform array; it is an almost-periodic one. Statistical analysis of the radiation pattern for imperfect array is then conducted to demonstrate that radiating main-beam angle (ensemble average) is dominated by the average period of a non-uniform array subject to a small perturbation. Additionally,the leaky wave of higher-order space harmonic in a periodic structure is employed to accurately predict the directional radiation including main beam as well as grating lobes.

## Introduction

In holography, the interference pattern, which is due to the superposition of object- and reference-waves, is recorded in a hologram. Once the reference wave is fed into the hologram, an object wave is emerging out of the structure.

In microwave or millimeter wave, the interference pattern can be implemented on dielectric substrate or metal layer using chemical etching^1,2^. However, the pattern is fixed and cannot be altered. Recently, a physical material, termed as metamaterial or meta-surface^3–7^ composed of subwavelength unit cell has been studied extensively. Moreover, some of the structures can even dynamically change its electrical properties by electric- or magnetic-bias. To mention a few: a dynamically reconfigurable holographic meta-surface aperture consisting of an array of subwavelength slot-shaped meta-elements was reported^8,9^. By launching a surface wave along waveguide whose impedance elements are rectangular patches with electrically tunable capacitors between them, a electronically-steerable, artificial-impedance-surface antenna was achieved^10,11^. A reconfigurable Metallic Electromagnetic Band-Gap antenna with controllable beam and directivity was proposed^12^. A reflection-type programmable metasurface composed of digital meta-atoms each integrated with a PIN diode for electronic control is developed^13^. The transmission-type 2-bit programmable coding metasurface for single-sensor and single-frequency imaging in microwave frequency was implemented^14^. A metamaterial antenna composed of electrically-small resonators was reported^15^.

Regarding dynamically reconfigurable holographic antennas design, the object wave is the desired beam directing at a prescribed direction while the reference wave is a waveguide mode. Each interference pattern (fringes) can be digitized and stored in a reconfigurable holographic meta-surface or metamaterial. By feeding a reference wave into the prescribed dynamically reconfigurable hologram, the beam steering can be realized. Consider the design method of such a class of antennas, a set of analytical formulations and numerical simulation for waveguide-fed meta-surface antennas was reported^16^. A discrete-dipole approximation model of a waveguide-fed planar metamaterial antenna is developed to accurately predict the radiation property of a two-dimensional metamaterial antenna^17^. Additionally, a systematic approach to synthesize the aperture field with analytical formulas is demonstrated in modulated-metasurface antennas capable of amplitude, phase and polarization control^18^. More specifically, the paper^16^ dealt with a waveguide-fed metasurface antenna including an interesting example of binary hologram with subwavelength on/off switchable slot-shaped unit cell. The states of “on” and “off” represent coupling or no-coupling, respectively.

Our research is based on the physical model developed in the work^16^. However, emphasis will be placed on system-level evaluation rather than on electromagnetic-field simulation for a real antenna structure. Generally speaking, such a class of antennas can be conceived as a waveguide (or transmission line) equipped with meta-atoms periodically arranged along the waveguide axis; each meta-atom can be independently controlled to couple some of power to radiate (on-state^16^) or to inhibit the coupling (off-state^16^), enabling a reconfigurable binary meta-hologram. The function of meta-atom is twofold: (1) to extract power from the guided mode, and (2) to radiate the coupled power into free space. The amplitude fed to each meta-atom for radiation is a complex number; its phase is determined by the propagation delay of the waveguide mode, while its strength relates to coupling coefficient that couples the guided-mode to meta-atom for radiation. Once the complex amplitude at each subwavelength radiator is known, the antenna array factor can be employed to calculate far-field radiation pattern.

Having the basic parameters including effective refractive index of the guided mode in a waveguide, spacing between two adjacent meta-atoms, and coupling coefficient, we are now in a good position to evaluate the radiation property of such a class of holographic metamaterial antennas. Here, two assumptions are made^16^ containing (1) the mutual coupling between meta-atoms is negligible, and (2) the meta-atom does not perturb the waveguide mode. Significantly, the design criterion for choosing effective radiating meta-atoms is developed to achieve a maximum value of array factor at a specific direction (angle). It is worth to note that the obtained lattice pattern is not always periodic; it is almost-periodic having small perturbation on their individual lattice spacing. Furthermore, the statistical analysis of the radiation main-beam angle against non-uniform lattices is carried out to demonstrate that the average spacing dominates the main-beam angle.

Additionally, the leaky wave due to the higher-order space harmonics^19^ in a periodic structure consisting of multiple discontinuities can be used to account for directional radiation pattern. The leaky-wave phase constant, which is less than *k*_*o*_ (free-space wavenumber), can be approximated by the higher-order space harmonic of guided-mode, while the leaky constant is related to the aforementioned coupling coefficient. Interestingly, each of the leaky waves corresponds to the main-beam or side-lobe calculated by array factor approach.

## Physical model and method of mathematical analysis

### Interference fringes in holography

Let’s begin with optical holography. Here we model the reference wave using a guided-wave with phase constant, *k*_*x*_ = *k*_*o*_*n*_*eff*_. The object wave is a plane wave incident at *θ*_*s*_. If the two waves share the same polarization and *k*_*y*_ = 0 (uniform field along the *y*-axis); the superposition of two waves is written as:

1 $$\phi (x)=\alpha {e}^{-j{k}_{o}{n}_{eff}x}+{E}_{o}{e}^{-j{k}_{o}\sin {\theta }_{s}x}=\alpha {e}^{-j{k}_{o}{n}_{eff}x}\left[1+\frac{{E}_{o}}{\alpha }{e}^{-j{k}_{o}x({n}_{eff}-\sin {\theta }_{s})}\right]$$

The interference pattern, in fact, is a continuous function with variable *x*. However, the feasible way to implement in microwave or millimeter-wave is the binary-amplitude hologram; that is, only the on/off states of the pattern are considered. For instance, the interference fringes with maximum |*ϕ*(*x*)| occurs at:

2 $$x({n}_{eff}-\,\sin \,{\theta }_{s})=p2\pi $$

where *p* belongs to integer. If the field along the *y*-axis is uniform, the interference pattern will be a collection of parallel lines with zero width along the *x*-axis.

Again, we relax the constrain to accommodate phase error, the above equation becomes:

3 $$p2\pi -\delta \psi /2\le x({n}_{eff}-\,\sin \,{\theta }_{s})\le p2\pi +\delta \psi /2.$$

Parameter *δψ* is defined as the phase error tolerance.

**Figure 1:**
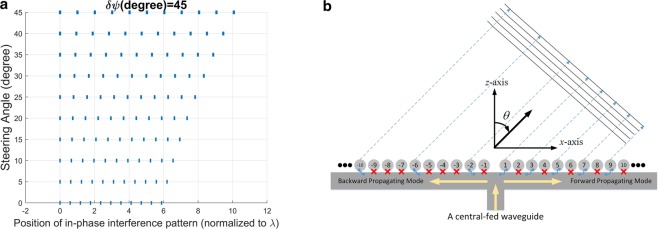
(**a**) Constructive interference pattern for various steering angles, and (**b**) schematic diagram of a 1D reconfigurable holographic antenna.

The position of in-phase interference fringes with *δψ* = *π*/4 along the *x*-axis is shown in Fig. 1(a). The width of each line along the *x*-axis increases in accordance with the increase in *δψ*. Apparently, each pattern corresponding to elevation angle (*θ*_*s*_) has its own period.

### Model a reconfigurable holographic antenna using a phased-array antenna

Figure 1(b) shows the schematic diagram of a reconfigurable holographic antenna for system-level simulation. A central-fed waveguide (shown as T-shaped one) generates forward- and backward-waves propagating along the right-hand side (RHS) and left-hand side (LHS), respectively. The ball symbol represents the meta-atom; for example, the subwavelength slot-shaped unit cell^16^. Moreover, the meta-atom is on/off switchable; the “on” state (through symbol in blue color) means that it can couple guided mode to meta-atom while “off” state (in red cross-shaped symbol) represents isolated between waveguide mode and meta-atom. As shown in this figure, the 1D array patterns are distinct in both sides. However, as will be clear later on, they can be individually designed to form a beam toward the same angle (direction).

### Phased array antennas

From array antenna theory^20–22^, the transmit/receive far-field pattern due to a one-dimensional (1D) uniform array antennas (sensors) can be simply determined by a superposition of each element radiation pattern. Once the mutual coupling is neglected, the overall pattern equals to the multiplication of array factor and single element pattern; where the array factor is written below:

4 $$AF(\theta ,\phi )=\mathop{\sum }\limits_{n=1}^{N}\,{\zeta }_{n}{e}^{+j\underline{k}\cdot {\underline{r}}_{n}}$$

where parameter *ζ*_*n*_ is the complex amplitude of each element at position ${\underline{r}}_{n}=\hat{x}{x}_{n}+\hat{y}{y}_{n}$, respectively; parameter $\underline{k}$ is the plane-wave propagating vector in free space. In rectangular coordinate system, we have $\underline{k}=\hat{x}{k}_{o}\,\sin \,\theta \,\cos \,\phi +\hat{y}{k}_{o}\,\sin \,\theta \,\sin \,\phi +\hat{z}{k}_{o}\,\cos \,\theta$, where *θ* is the elevation angle counted from the *z*-axis; parameter *ϕ* is the azimuth angles counted from the *x*-axis, respectively. Here we assume that the array elements are over the *x*-*y* plane.

### Array factor in forward-propagating scenario

Let’s consider a series-fed 1D array with uniform period denoted as *d*_*x*_. The complex amplitude at the *n*^*th*^ antenna, *x* = *x*_*n*_, can be written as ${\zeta }_{n}={\alpha }_{n}{e}^{-j\beta |{x}_{n}|}$, where *α*_*n*_ is the amplitude and *β|x*_*n*_*|* is the propagation phase of guided-wave, with phase constant denoted as *β*, traveling to *x* = *x*_*n*_; *x* = 0 is the initial position of the waveguide. Therefore, Eq. (4) can be rewritten as:

5 $$AF(\theta )=\mathop{\sum }\limits_{n=0}^{N-1}\,{\alpha }_{n}{e}^{-j\beta |{x}_{n}|}{e}^{+j{k}_{o}{x}_{n}\sin \theta }$$

where $${x}_{n}={x}_{o}^{(+)}+n{d}_{x}$$.

By substituting *β* = *k*_*o*_*n*_*eff*_ into Eq. (5), where *n*_*eff*_ is the effective refractive index of guided mode in the waveguide, we obtain:

6 $$AF(\theta )=\mathop{\sum }\limits_{n=0}^{N-1}\,{\alpha }_{n}{e}^{-j{k}_{o}({n}_{eff}|{x}_{n}|-{x}_{n}\sin \theta )}={e}^{-j{k}_{o}({n}_{eff}-\sin {\theta }_{s}){x}_{o}^{(+)}}\mathop{\sum }\limits_{n=0}^{N-1}\,{\alpha }_{n}{e}^{-jn{k}_{o}({n}_{eff}-\sin \theta ){d}_{x}}$$

The absolute value of array factor is given below:

7 $$|AF(\theta )|=|\mathop{\sum }\limits_{n=0}^{N-1}\,{\alpha }_{n}{e}^{-jn{k}_{o}({n}_{eff}-\sin \theta ){d}_{x}}|$$

If we would have a global maximum taking place at *θ* = *θ*_*s*_, where *θ* belongs to [−*π*/2, +*π*/2], the condition of constructive interference must be met:

8 $$n{k}_{o}{d}_{x}({n}_{eff}-\,\sin \,{\theta }_{s})=p2\pi $$

where *p* belongs to integer including zero. The index *n* is obtained as:

9 $$n=\frac{p2\pi }{{k}_{o}{d}_{x}({n}_{eff}-\,\sin \,{\theta }_{s})}$$

For the discrete points (${x}_{n}={x}_{o}^{(+)}+n{d}_{x}$) along the *x*-axis, the index in Eq. (9) must be an integer. However, let’s look at its right-hand side, it is not always possible. In fact, some tolerance in the phase error is allowed to maintain a global maximum at *θ*_*s*_. We may modify the constrain in Eq. (8) as:

10 $$p2\pi -\delta \psi /2\le n{k}_{o}{d}_{x}({n}_{eff}-\,\sin \,{\theta }_{s})\le p2\pi +\delta \psi /2$$

where *δψ* is defined as the phase-error tolerance for each element to direct its maximum strength toward *θ*_*s*_. Therefore, the index *n* is in a range given below:

11 $$\frac{\lambda }{{d}_{x}}\frac{p-\delta \psi /4\pi }{{n}_{eff}-\,\sin \,{\theta }_{s}}\le n\le \frac{\lambda }{{d}_{x}}\frac{p+\delta \psi /4\pi }{{n}_{eff}-\,\sin \,{\theta }_{s}}$$

In the above equation, for each index *p* starting from zero, we may find a corresponding element with its position within the range given in Eq. (11).

### Array factor in backward-propagating scenario

Let’s consider a backward wave propagating from *x* = 0 along negative *x*-axis, shown in Fig. 1(b) for the LHS 1D array. The discrete points for extracting electromagnetic field are allocated at ${x}_{m}={x}_{o}^{(-)}+(-m){d}_{x}$, where *m* is an integer running from zero to *M*. Apply the same procedure for evaluating the array factor, we obtain:

12 $$AF(\theta )=\mathop{\sum }\limits_{m=0}^{M-1}\,{\alpha }_{m}{e}^{-j{k}_{o}({n}_{eff}|{x}_{m}|-{x}_{m}\sin \theta )}={e}^{j{k}_{o}({n}_{eff}+\sin {\theta }_{s}){x}_{o}^{(-)}}\mathop{\sum }\limits_{m=0}^{M-1}\,{\alpha }_{m}{e}^{jm{k}_{o}({n}_{eff}+\sin \theta ){d}_{x}}$$

Similarly, to have a global maximum at around *θ*_*s*_, with a tolerance in phase error, we have the constrain given below:

13 $$q2\pi -\delta \psi /2\le m{k}_{o}{d}_{x}({n}_{eff}+\,\sin \,{\theta }_{s})\le q2\pi +\delta \psi /2$$

where *q* is an integer starting from zero.

Therefore, the index *m* is in the range written below:

14 $$\frac{\lambda }{{d}_{x}}\frac{q-\delta \psi /4\pi }{{n}_{eff}+\,\sin \,{\theta }_{s}}\le m\le \frac{\lambda }{{d}_{x}}\frac{q+\delta \psi /4\pi }{{n}_{eff}+\,\sin \,{\theta }_{s}}$$

## Beamforming of a end-fed holographic metamaterial antenna

Here, we give an example to illustrate the theory developed previously. Consider a guided mode propagating in a uniform waveguide with effective refractive index *n*_*eff*_  = 1.7(*k*_*x*_  =  *k*_*o*_*n*_*eff*_) operated at *λ* = 10 *mm*. The spacing between any two adjacent meta-atoms is *d*_*x*_  = 1.2 *mm*, which is much smaller than the operating wavelength. Notably, the seamless scan in radiation pattern is impossible because of discrete meta-atoms. The change in the period of a binary synthetic meta-hologram is absolutely not continuous.

Furthermore, we consider two scenarios with the guided-wave propagating along forward (+*x*) and backward (−*x*) directions both. The distance from fed point to the first meta-atom is *d*_*x*_. The desired radiating main-beam angle is designated as *θ*_*s*_, which is counted from the *z*-axis shown in Fig. 1(b). Substitution of the structure parameters into Eq. (11), we may determine a corresponding lattice topology having a directional pattern along *θ*_*s*_ direction. Notice that the phase-error tolerance *δψ* is set to be *π*/6 in this example.

**Figure 2:**
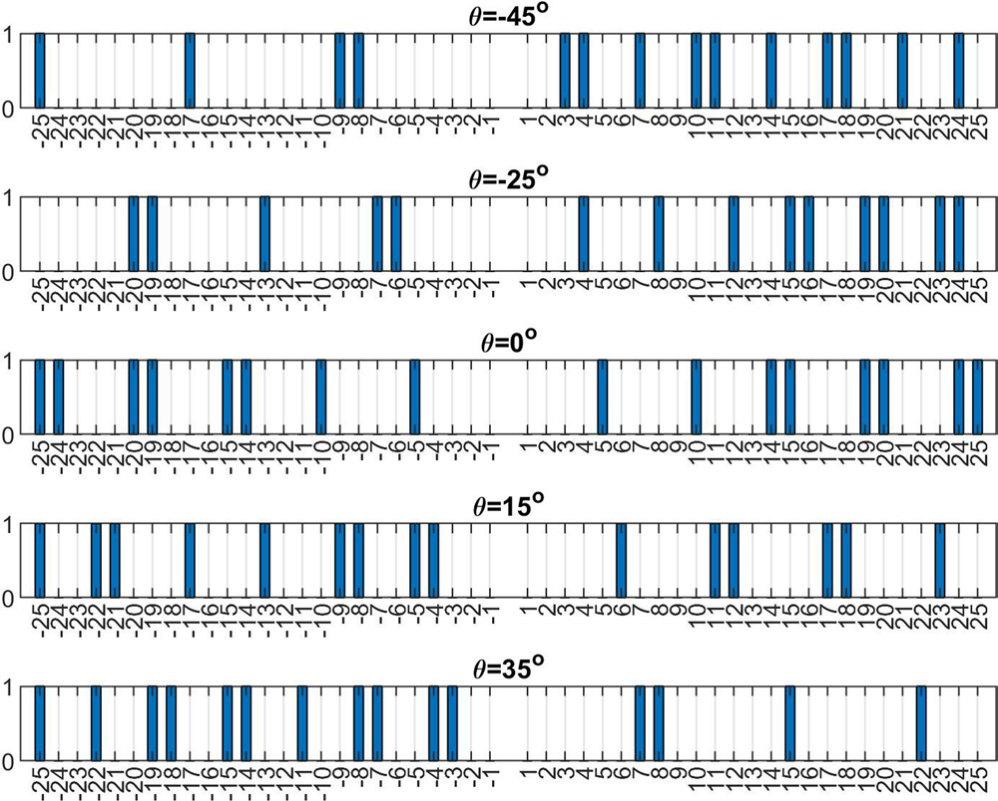
Array on-off state corresponding to each steering angle with *d*_*x*_ = 1.2 *mm*; positive index for forward-wave excitation and negative index for backward-wave; “1” is for “on”-state and “0” for “off”-state.

Figure 2 shows the on/off state of each meta-atom, corresponding to various radiating main-beam angles. Symbols “1” and “0” represent “on” and “off” state, respectively. The positive index number refers to the 1D array fed by forward propagating wave, while negative ones are designated for that excited by backward wave.

Notably, the successive lattice points are chosen to approximate a non-integer one in the range of Eq. (11). For example, if *n* belongs to [5.3, 5.8], it does not contain any integer number; nevertheless, we can not neglect it therein. Consequently, we put both lattice points: *n* = 5 and *n* = 6 to approximate the non-integer one. Moreover, the obtained lattice might be non-uniform; in fact, they are almost-periodic structure having small perturbation on their spacing between two adjacent lattice points.

**Figure 3:**
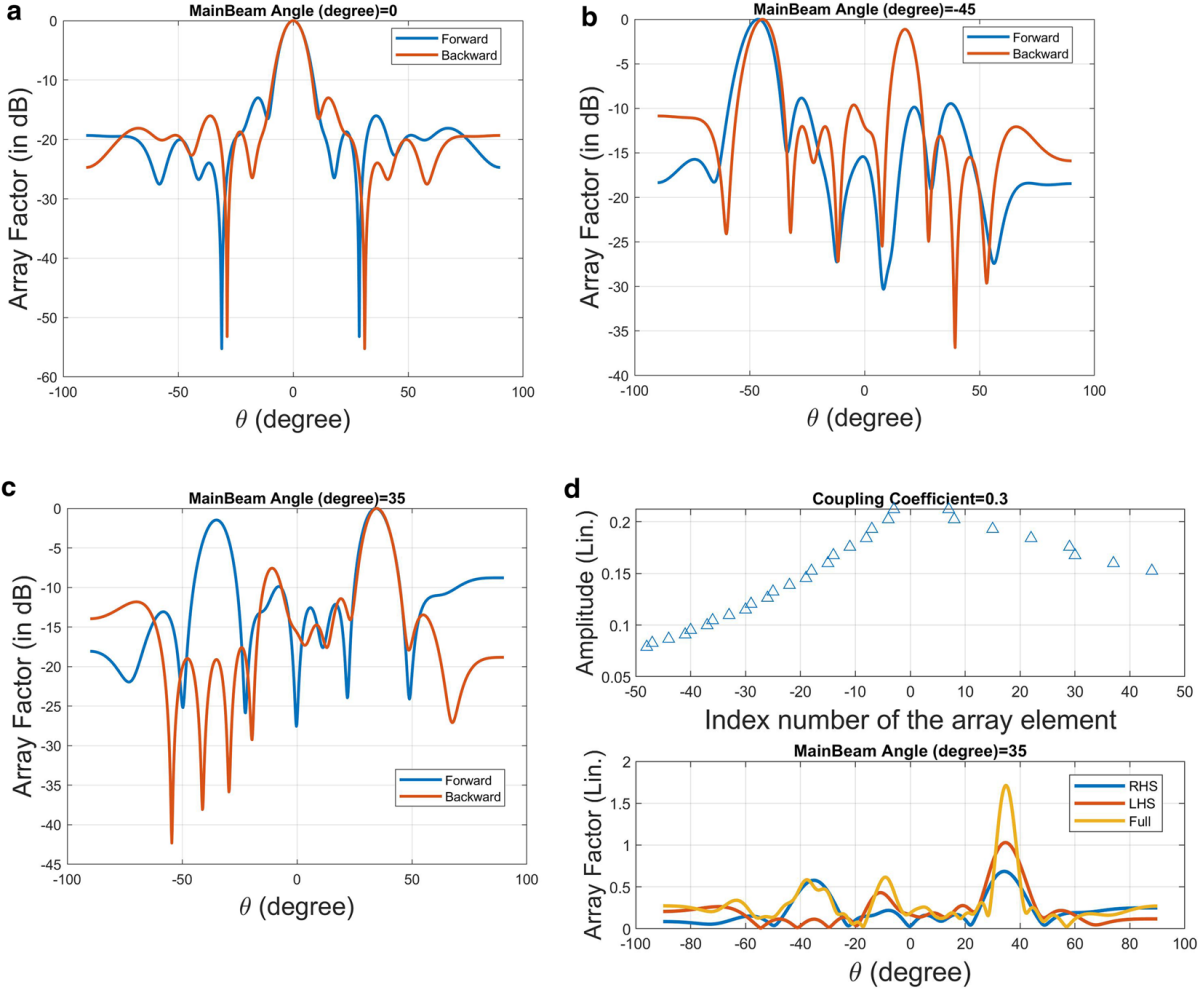
Array factor against radiation angle (*θ*) for the binary meta-hologram listed in Fig. 2 excited by the forward- and backward-waves; the radiation main-beam angle for each case is respectively designed at (**a**) *θ* = 0°, (**b**) *θ* = −45°, (**c**) *θ* = 35°, and (**d**) the amplitude distribution at each element along both the RHS and LHS arrays in case (**c**) is shown together with their individual (blue color for RHS and red for LHS) and superposition (in orange color) of radiation far-field pattern expressed in linear scale.

Not only the lattice pattern, we also calculate the array factor corresponding to these lattice patterns. Figure 3(a) shows the array factor against *θ* for the lattice with pattern denoted as *θ* = 0*°* in Fig. 2. Both lattices in the LHS and RHS share the same pattern; the average period of the almost-periodic structure is around 5.8667 *mm*. Apparently, the main-beam angle is as expected to be at 0 degree.

Figure 3(b) demonstrates the array factor for the first row in Fig. 2; the blue curve is forward-wave excitation with average period around 4.1538 *mm* and red one corresponds to backward wave with average period about 10.05 *mm*. Both cases are designed to form directional beam pointing at around −45*°*, shown in this figure. Nevertheless, the grating lobe appears for the backward-wave case since its average period is larger than operating wavelength.

In Fig. 3(c), corresponding to the last row in Fig. 2, the average periods of the 1D arrays excited by forward- and backward-waves are 8.88 *mm* and 4.4 *mm*, respectively. Contrary to the previous example, the grating lobe appears in the forward case. As is well known in antenna theory, the period greater than half operating wavelength may cause grating lobes, in particular for the main-beam is near the end-fire direction. In the last section of this paper, theory of leaky-wave in a periodic structure will be exploited to elaborate on this issue again.

Interestingly, the radiation main-beam angle indeed corresponds to the designed ones listed in the Fig. 2, although they are not perfect arrays with equal separation distances. In the following section, we will investigate the effect of imperfect lattice, particularly for the non-uniform period, on the radiating main-beam angle based on statistical analysis.

## Sensitivity analysis of imperfect 1D arrays

In this section, a 1D array having slightly non-uniform separation distance between any two adjacent lattice points was investigated. Each of the separation distance is expressed as a random variable with a mean denoted as *μ* and a standard deviation designated as *σ*, which is written below:

15 $$[{d}_{x}]=\mu +\sigma [\zeta ]$$

Period *d*_*x*_ is a random variable, where *ζ* is a normally distributed pseudo-random number. We may generate a large number of 1D lattice patterns, each having *N* lattice points and *N* − 1 spacings, given by Eq. (15). Substituting them into Eq. (6), we may determine the main beam angle by searching the peak-value position of the array factor.

**Figure 4:**
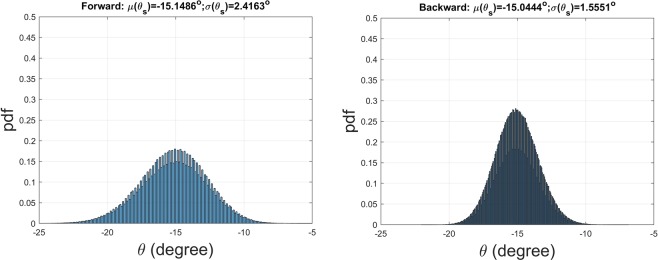
Statistical analysis of the far-field main-beam angle (**a**) forward-wave excitation, and (**b**) backward-wave excitation; the number of samples (lattice patterns) equals to one million. The binary meta-hologram corresponding to (**a**,**b**) are given in the column entitled *θ* = 15° of Fig. 2.

The first example to be demonstrated is a 1D imperfect lattice consisting of 11 meta-atoms excited by a forward wave. The mean and standard deviation of spacing are *μ* = 5.1 *mm* and *σ* = 0.3162 *mm*, respectively; the coupling coefficient is *X*_*c*_ = 0.3. Those parameters correspond to the design, which is based on Eq. (11), of a non-uniform lattice with the main beam directing at *θ* = −15*°*. The histogram presented in the format of probability density function (pdf) is shown in Fig. 4(a), with the number of samples equal to one million.

In the second example depicted in Fig. 4(b), the backward-wave is fed into the 1D imperfect lattice with *μ* = 6.9429 *mm* and *σ* = 0.3207 *mm*, respectively. The number of meta-atoms is 8. Obviously, the mean of main-beam angle is very close to the designed one at −15*°*. Furthermore, the standard deviation is smaller than the previous case since the level of perturbation (defined as the ratio of *σ* to *μ*) in the later case is smaller than that of the former one.

## Leaky-wave viewpoint

Rigorous formulation on guiding characteristics of surface wave on an impedance surface having a sinusoidally modulated reactance can be traced back to the research work^23^, which lays a solid foundation for understanding the guided and leaky-waves supported by an artificial impedance surface (or meta-surface in today’s terminology). The surface-impedance model is also utilized to successfully explain Wood’s anomalies on optical gratings^24^. Even the wave guiding and scattering by a two-dimensional anisotropic periodic impedance surface has also been formulated rigorously^19^.

A waveguide having periodic discontinuities can radiate its power into surrounding medium^19^, enabling a directional (pencil) beam pointing at a specific angle that can be predicted by its guided-wave phase constant (*β*) and period (*D*) given below:

16 $${k}_{o}\,\sin \,{\theta }_{n}=\pm \beta +n\frac{2\pi }{D}$$

Where index *n* is an integer running from negative- to positive-infinity including zero. The ± sign in *β* means the wave is propagating along forward- or backward-direction. Parameter *D* is the period of a 1D grating. If we have *β*  =  *k*_*o*_*n*_*eff*_, therefore, Eq. (16) can be rewritten as:

17 $${\theta }_{n}={\sin }^{-1}\left(\pm {n}_{eff}+n\frac{\lambda }{D}\right).$$

where *n*_*eff*_ is the effective refractive index of the guided mode.

Moreover, *n* is an integer and is termed as the index of space harmonic or Floquet mode in a periodic structure. There are an infinite number of space harmonics in an infinite periodic structure; however, only a finite numbers of them can be radiating (or leaking) into the surrounding medium; the other are non-propagating (evanescent) waves. Each leaky (propagating) wave corresponds to a plane wave with a definite radiating main-beam angle and can be determined by Eq. (17). Specifically, the number of propagating space harmonic (in free space) can be determined by the condition given below:

18 $$|\sin \,{\theta }_{n}|=|\pm \beta /{k}_{o}+n\frac{\lambda }{D}|\le 1$$

For a backward propagating wave with *β* = −*k*_*o*_*n*_*eff*_, its space harmonic indices are positive integers in the range given below:

19 $$\frac{D}{\lambda }({n}_{eff}-1)\le n\le \frac{D}{\lambda }({n}_{eff}+1).$$

Contrarily, for a forwarding propagating wave having *β* = +*k*_*o*_*n*_*eff*_, its space harmonic indices are negative integers in the range:

20 $$-\frac{D}{\lambda }(1+{n}_{eff})\le n\le \frac{D}{\lambda }(1-{n}_{eff}).$$

Consider a forward wave propagating along a 1D periodic waveguide having period *D*_*x*_  = 4.1538 *mm* with effective refractive index *n*_*eff*_  = 1.7 at 30 *GHz*. We may find the main-beam angle via Eq. (17). Specifically, because of *λ*/*D* = 2.4074, only the index *n* = −1 satisfies |1.7 + 2.4074*n*| ≤ 1.0 in Eq. (18). The main-beam angle is *θ*_*n=*−1_  = −45.0238*°*. On the other hand, if a backward-wave is propagating along negative *x*-axis with the same *n*_*eff*_, the radiation main-beam angle will be sin^−1^(−1.7 + *n*0.9950) subject to *D* = 10.05 *mm*. Here, two indices: *n* = +1 and *n* = +2 represent two grating lobes. The first one (*n*  = +1) corresponds to *θ*_*n=*+1_  = −44.8295*°*; while the second one radiates to *θ*_*n=*+2_ = 16.858*°*, respectively. Those two space harmonics correspond to the main beam and grating lobe, which can be confirmed in Fig. 3(b).

Additionally, we again carry out the radiation pattern sensitivity analysis for several 1D imperfect lattices. The mean and standard deviation of the main-beam angle for each case were listed in Table 1. It is apparent to see that the mean angle agrees with the prescribed one. The columns denoted as **F**(forward-wave excitation): *θ*_*LW*_ and **B**(backward-excitation): *θ*_*LW*_ are the main-beam angles estimated by Eq. (17) based on the leaky-wave viewpoint. The agreement between them looks excellent for every case.

**Table 1:** Sensitivity analysis on the radiation main-beam angle against the non-uniform lattice; number of samples = 100000.

*θ*(^*o*^)	F: *μ*(^*o*^)/*σ*(^*o*^)	F: *θ*_*LW*_(^*o*^)	B: *μ*(^*o*^)/*σ*(^*o*^)	B: *θ*_*LW*_(^*o*^)
−45	−45.0708/2.3171	−45.0244	−44.8265/1.2599	−44.8275
−30	−31.5479/3.2193	−31.4814	−29.6837/0.9911	−29.6817
−15	−15.1567/2.4248	−15.1166	−15.0466/1.5613	−15.0505
0	−0.2739/1.5446	−0.2604	0.2668/1.5439	0.2604
15	15.0504/1.5517	15.0505	15.1406/2.4148	15.1166
30	29.6794/0.9891	29.6817	31.5852/3.2385	31.4814
45	44.8313/1.2741	44.8275	45.0923/2.3270	45.0244

## Beamforming of a central-fed holographic metamaterial antenna

As detailed in previous sections, two different 1D periodic structures along the +*x* and −*x* directions can radiate (leak) the feeding signal to the same direction, as shown in Fig. 3(a–c). Let’s consider a waveguide fed at the central position; thus, forward- and backward-propagating modes are excited shown in Fig. 1(b). The superposition of two complex array factors in Eqs. (6) and (12) shall enhance the radiation along the prescribed direction.

Figure 3(d) shows array factors of a central-fed waveguide having two distinct lattice patterns along ±*x*-direction, which are depicted in the last row of Fig. 2, respectively. The upper figure shows the amplitude at each meta-atom to be radiated. Because the input power is gradually radiating by the successive elements, amplitude tapers can be found. In the lower figure, the array factor contributed by both right-handed and left-handed sides indeed enhance the radiation at around *θ* = 35*°*. Apparently, the grating lobe due to the right-handed-side lattice may deteriorate the overall radiation pattern. Regarding grating-lobes and sidelobes reduction, an efficient and robust algorithm for optimizing performance of an holographic metamaterial antenna was reported^25,26^.

**Figure 5:**
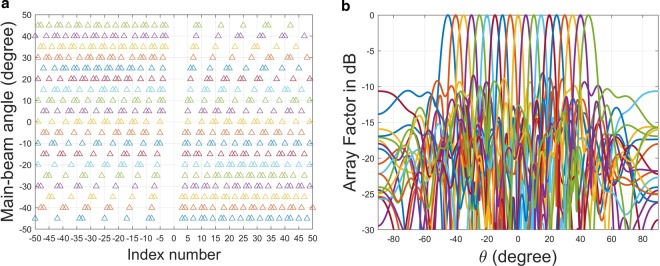
Beam-steering characteristics of a reconfigurable 1D holographic metamaterial antenna: (**a**) On/off of the 1D lattice versus radiating main-beam angle: *d*_*x*_  = 1.2 *mm*, *λ*  = 10 *mm*, *X*_*c*_  = 0.3, ${x}_{o}^{+}={d}_{x}$, ${x}_{o}^{-}=-{d}_{x}$, *n*_*eff*_  = 1.7, (**b**) Array factor versus *θ*.

The last example is to demonstrate the beam-steering characteristics of the reconfigurable holographic antenna. A central-fed waveguide equipped with 100 on/off switchable meta-atoms is considered. The on/off state of each meta-atom corresponding to the prescribed radiating main-beam angle has been arranged as a lookup table and is shown in Fig. 5(a), with the triangle symbol indicates the on-state.

We calculate the array factor of each array shown in Fig. 5(a). In Fig. 5(b), the main-beam angle is scanning from −45*°* to +45*°* once the lattice pattern is changing from the last row to the first row in Fig. 5(a). Due to the binary scheme in the coupling coefficient, the side lobe level is relatively high. However, it can be further suppressed by incorporating the sophisticated coupling coefficient.

## Numerical experiment

**Figure 6:**
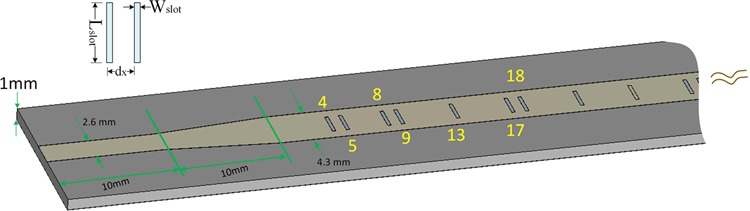
Structure configuration of the holographic metamaterial antenna for numerical experiment (partial structure shown). The index number attached on the slot corresponds to “on”-state. The unit cell of the metamaterial structure consists of a radiating slot, which is attached as a small inset. The structure parameters are given below: substrate thickness: 1 *mm*, unit-cell length: *d*_*x*_ = 1.2 *mm*, slot width: *W*_*slot*_ = 0.3 *mm*, slot length: *L*_*slot*_ = 2.3 *mm*; strip-line width: 4.3 *mm*. The input/output micro-strip line width and length are 2.6 *mm* and 10 *mm*, respectively; taper-transition length is 10 *mm*.

**Figure 7:**
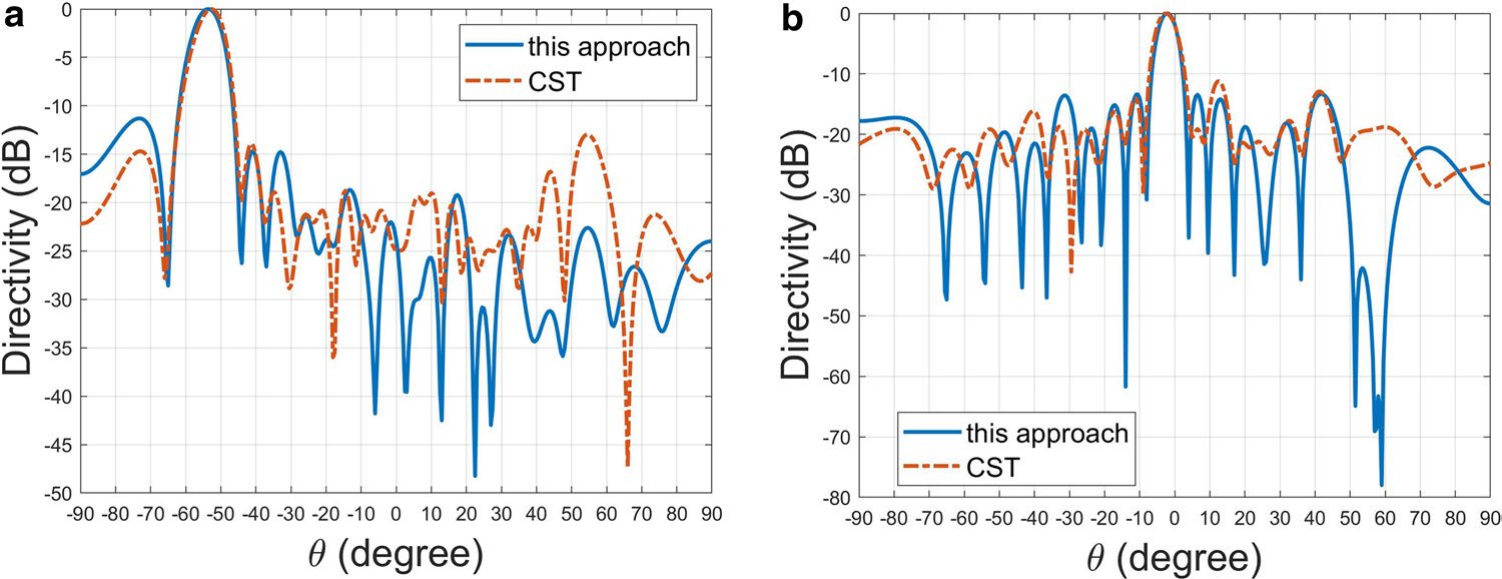
Directivity (in linear scale) on *x*-*z* plane against *θ* for the two antennas with different binary meta-hologram pattern evaluated at 29.5 *GHz*; (**a**) the indices of “on”-state slots are [3 6 9 12 13 15 16 18 19 21 22 24 25 27 28 30 31 33 34 37 40 43 46 49 50 52 53 55 56 58 59 61 62 64 65 68 71 74 77 80], (**b**) the indices of “on”-state slots are [4 5 8 9 13 17 18 22 26 30 31 35 39 40 43 44 48 52 53 57 61 65 66 70 74 75 78 79].

To validate the design theory developed here, numerical experiments were carried out using a commercial, full-wave 3D electromagnetic simulation software (CST Microwave Studio) based on time-domain finite integration technique. Figure 6 shows a holographic metamaterial antenna made of a micro-strip line with slot-shaped unit cells on its front face^16^. To reduce the impedance mismatch between the antenna and feed line, taper-transition structures are implemented. An ultra-low-loss dielectric slab with dielectric constant (Dk) 3.5 (IT-8350A, ITEQ Corporation) was used to serve as PCB (printed circuit board) substrate. The position corresponding to “on” state is equipped with a slot for converting guided-mode into leaky-wave. We design two meta-hologram patterns with their main-beam angle directing to distinct angles. The micro-strip line mode has *n*_*eff*_  = 1.81 at 29.5 *GHz*; each slot introduces an extra phase delay angle around 12*°*. The coupling coefficient is *X*_*c*_  = 0.16. As shown in Fig. 7(a,b), apparently, in the vicinity of main beam the result of analytical calculation agrees with that of numerical simulation for both cases. However, the sidelobes cannot be correctly estimated; it may be conjectured that this approach merely calculates the array factor without considering radiation pattern of individual slot and mutual coupling.

## Conclusion

In this research, a 1D reconfigurable holographic antenna is modeled as a series-feeding 1D phased array with their signal source provided by a guided mode propagating in a waveguide through subwavelength on/off switchable meta-atoms. Thus, the phase and amplitude relate to the propagation constant of the guided-mode and coupling coefficient, respectively. Consequently, a simple mathematical formulation based on array factor is employed to evaluate far-field radiation pattern. Significantly, a design criterion for generating the binary meta-hologram pattern that radiates a prescribed beam pattern is developed. Notably, the obtained array is not always periodic; in fact, it is almost-periodic (imperfect lattice) one. The array-factor statistical analysis was carried out to prove that the ensemble average of main-beam angle depends on the average period subject to a small perturbation. Alternatively, the structure under consideration, in fact, is a grating waveguide with periodic discontinuities simultaneously scattering its guided-wave energy into the surrounding medium. Therefore, the Floquet-Bloch theory is successfully applied to correctly predict leaky-wave angles including main-beam and grating lobes. Additionally, numerical experiments have also been conducted to validate the design principle developed in this paper.
